# Structural and functional understanding of disease-associated mutations in V-ATPase subunit a1 and other isoforms

**DOI:** 10.3389/fnmol.2023.1135015

**Published:** 2023-07-03

**Authors:** Karen Indrawinata, Peter Argiropoulos, Shuzo Sugita

**Affiliations:** ^1^Division of Translational and Experimental Neuroscience, Krembil Brain Institute, University Health Network, Toronto, ON, Canada; ^2^Department of Physiology, Faculty of Medicine, University of Toronto, Toronto, ON, Canada

**Keywords:** V-ATPase, subunit a1, R740, neurodevelopmental disorders, developmental and epileptic encephalopahties, progressive myoclonus epilepsy

## Abstract

The vacuolar-type ATPase (V-ATPase) is a multisubunit protein composed of the cytosolic adenosine triphosphate (ATP) hydrolysis catalyzing V_1_ complex, and the integral membrane complex, V_o_, responsible for proton translocation. The largest subunit of the V_o_ complex, subunit a, enables proton translocation upon ATP hydrolysis, mediated by the cytosolic V_1_ complex. Four known subunit a isoforms (a1–a4) are expressed in different cellular locations. Subunit a1 (also known as V_o_a1), the neural isoform, is strongly expressed in neurons and is encoded by the *ATP6V0A1* gene. Global knockout of this gene in mice causes embryonic lethality, whereas pyramidal neuron-specific knockout resulted in neuronal cell death with impaired spatial and learning memory. Recently reported, *de novo* and biallelic mutations of the human *ATP6V0A1* impair autophagic and lysosomal activities, contributing to neuronal cell death in developmental and epileptic encephalopathies (DEE) and early onset progressive myoclonus epilepsy (PME). The *de novo* heterozygous R740Q mutation is the most recurrent variant reported in cases of DEE. Homology studies suggest R740 deprotonates protons from specific glutamic acid residues in subunit c, highlighting its importance to the overall V-ATPase function. In this paper, we discuss the structure and mechanism of the V-ATPase, emphasizing how mutations in subunit a1 can lead to lysosomal and autophagic dysfunction in neurodevelopmental disorders, and how mutations to the non-neural isoforms, a2–a4, can also lead to various genetic diseases. Given the growing discovery of disease-causing variants of V-ATPase subunit a and its function as a pump-based regulator of intracellular organelle pH, this multiprotein complex warrants further investigation.

## 1. Introduction

The vacuolar H^+^-ATPase (V-ATPase) is an adenosine triphosphate (ATP)-dependent proton pump that regulates electrochemical gradients between membranes. These pumps are critical for pH homeostasis in the extracellular milieu and intracellular organelles, such as lysosomes, the Golgi network, and the endoplasmic reticulum (Wang L. et al., 2020). Membrane V-ATPases are crucial for various biological processes as they acidify specialized organelles involved in bone resorption (Toyomura et al., 2003), sperm maturation (Pietrement et al., 2006), and proton secretion by intercalated kidney cells (Wagner et al., 2004). V-ATPases are also involved in various signaling cascades, including Wnt, Notch, and mTOR, important for regulating cell proliferation and differentiation (Zoncu et al., 2011; Sun-Wada and Wada, 2015). Organellar V-ATPases establish and maintain the pH of endosomes and lysosomes for intracellular membrane trafficking and protein degradation (Forgac, 2007). V-ATPases are also suggested to play an important role in regulating membrane fusion and neurotransmitter release, such as glutamate and norepinephrine (Hinton et al., 2009). Furthermore, increased expression of V-ATPase on the plasma membrane facilitates cancer progression by maintaining an acidic tumor microenvironment (Pamarthy et al., 2018).

The V-ATPase is a large multisubunit rotary machine composed of two dissociable sectors: the peripheral V_1_ sector responsible for ATP hydrolysis and the membrane integral V_o_ sector governing proton translocation. Human V-ATPases consist of eight V_1_ subunits (A_3_, B_3_, C, D, E_3_, F, G_3_, H) and eight V_o_ subunits (a, c_9_, c″, d, e, Ac45, RNaseK and ATP6AP2), some of which are present in multiple copies as denoted by the subscript numbers. These subunits congregate and collaborate to hydrolyze ATP and subsequently conduct proton translocation across membranes (Wang L. et al., 2020). Subunit a is the largest of the V_o_ complex (~110 kDa) and mediates the entry and exit pathway of protons from the cytoplasmic to the luminal side, coupled with c-ring rotation powered by ATP hydrolysis (Kawasaki-Nishi et al., 2001b; Wang L. et al., 2020).

The V-ATPase pumps protons into the lumen of lysosomes, thereby decreasing the pH to activate autophagic enzymes. Disruption of neuronal lysosomes and their autophagic function cause aberrant accumulation of intracellular proteins and the formation of inclusion bodies, leading to neurodegeneration (Hara et al., 2006; Komatsu et al., 2006). Likewise, studies reported that dysfunction of the a1 subunit is implicated in neuronal impairment. For example, global knockout of the subunit a1 gene, *ATP6V0A1* (located on chromosome 17q21.2), leads to embryonic lethality (Dickinson et al., 2016). Furthermore, impeded V-ATPase assembly and function due to impaired subunit a1 glycosylation and stability upon mutations to presenilin-1 (PS-1) was observed in the Alzheimer’s disease (AD) mouse model (Lee et al., 2015). Analysis of variant frequencies in AD reported that PS-1 mutations are the most likely pathogenic variant, followed by mutations to the amyloid precursor protein (APP) and presenilin-2 (PS-2) respectively, highlighting the indirect relationship between subunit a1 and AD (Xiao et al., 2021). Moreover, misrouted a1 is the main cause of lysosomal dysregulation in the mouse model of infantile neuronal ceroid lipofuscinoses, also known as Batten disease (Bagh et al., 2017). a1 conditional knockout in the forebrain pyramidal neurons of mice also resulted in general brain atrophy of hippocampal CA1 and displayed impaired learning and memory (Ma et al., 2019). Recently, studies have reported that various *de novo* and biallelic mutations to *ATP6V0A1* are a major cause of DEE (developmental and epileptic encephalopathies) (Aoto et al., 2021; Bott et al., 2021). The R741Q mutation (R740Q based on the updated accession number NM_001130021.3) leads to impaired proton translocation and is a recurring DEE-causing variant. Additionally, a1 compound heterozygous variants, E149Kfs18 and R495W, are identified in early-onset PME (progressive myoclonus epilepsy) with ataxia (Bott et al., 2021). These findings demonstrate the crucial role of the a1 subunit in a functional V-ATPase for neuronal lysosome acidification and autophagosomes. It also highlights V-ATPases pathophysiology in neurodevelopmental and neurodegenerative disorders.

In this review, we will first cover the structure and mechanism of the V-ATPase, focusing on subunit a1. We will then discuss how various mutations to key a1 residues impair lysosomal and autophagosomal processes involved in neurodegenerative disease. Additionally, we will consider disease-associated variants of the other subunit a isoforms: a2, a3, and a4. Our review highlights the importance of understanding the pathophysiology of *ATP6V0A1* mutations. Further studies will improve the knowledge surrounding V-ATPase’s role in brain development and neurodevelopmental disorders with lysosomal and autophagic dysfunction.

## 2. Structure and function of the V-ATPase and subunit a1

**Figure 1:**
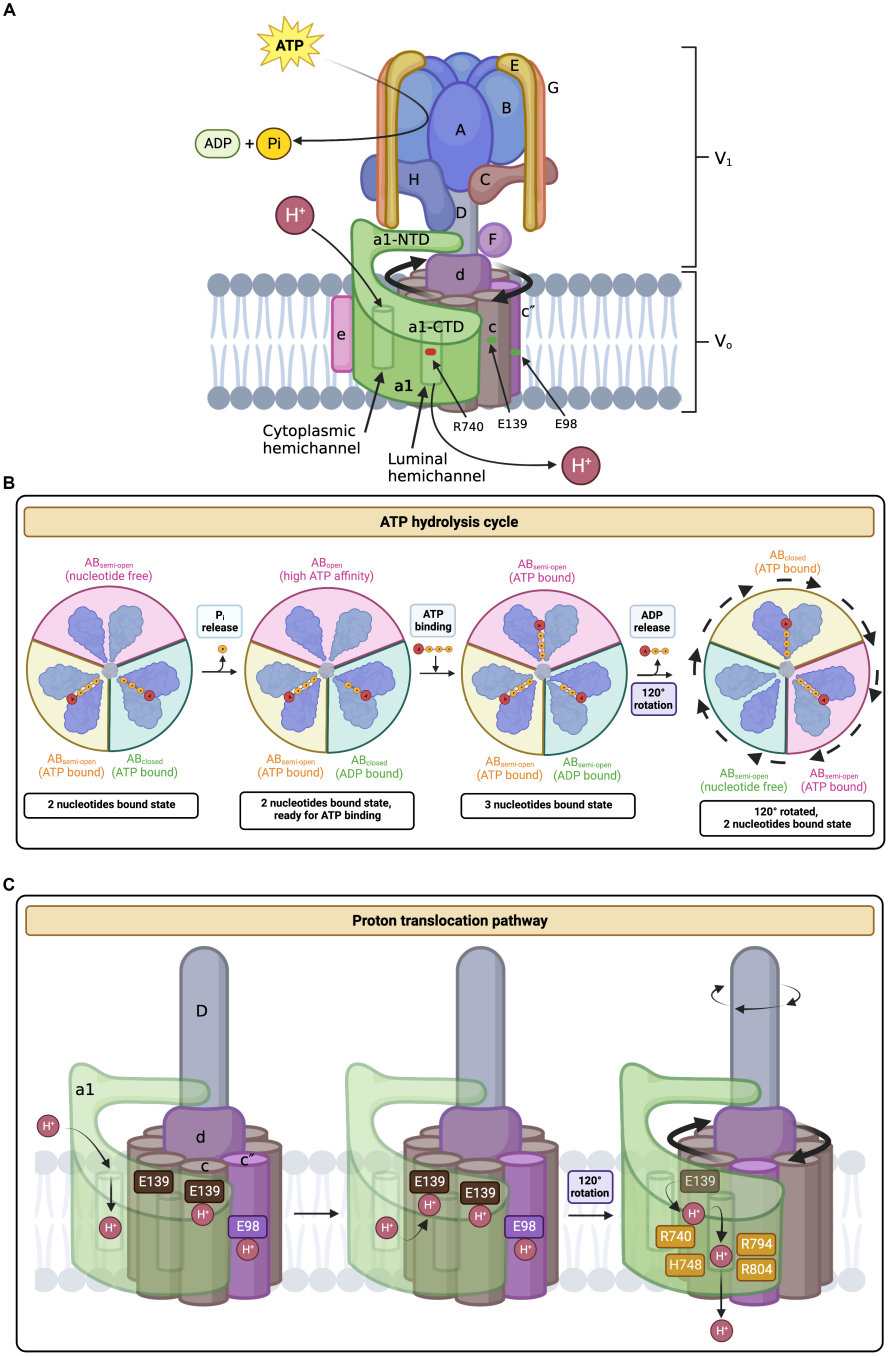
Structure and mechanism of the human V-ATPase. **(A)** V-ATPase structure. The cytoplasmic V_1_ sector is responsible for ATP hydrolysis and contains the following subunits: A_3_, B_3_, C, D, E_3_, F, G_3_, and H. The membrane-embedded V_o_ sector mediates proton translocation and is composed of the following subunits: a1 (neuronal isoform), c_9_, c″, d, e, Ac45 (also known as ATP6AP1), RNaseK, ATP6AP2. The top of V_1_ includes the A_3_B_3_ hexamer head, the site of ATP binding and hydrolysis. ATP hydrolysis induces the rotation of the A_3_B_3_ hexamer which subsequently powers the rotation of the c-ring (composed of subunits c_9_ and c″) via linkage through subunits D, F, and d (the central stalk). The peripheral stalks, composed of subunits E and G, act as stators and connect the V_1_ sector with V_o_. Protons enter and exit subunit a1 via the hemichannels and interact with key residues in a1 and the c-ring in the process of proton translocation. **(B)** ATP hydrolysis cycle. ATP hydrolysis occurs between the interface of the A and B subunits in the V_1_ complex. The C-terminal domains of A and B undergo conformational changes depending on their bound state to ATP and ADP. First, ATP is tightly bound between the A and B subunits and is subsequently hydrolyzed into ADP and inorganic phosphate (Pi). The Pi is released and the AB pair changes conformations to a less tight ADP-bound form. This triggers the adjacent AB pair to change conformations from a semi-open (nucleotide-free) conformation to a high ATP-affinity open confirmation. Next, ATP binds tightly to the open AB pair, changing the ADP-bound dwell into a semi-open conformation, thus releasing ADP. The conformational changes associated with the ATP hydrolysis cycle induce the tilting and oscillation of the A_3_B_3_ hexamer. **(C)** Proton translocation pathway. A proton enters the cytoplasmic hemichannel and protonates an essential glutamic acid residue on the c-ring (E139 subunit c or E98 subunit c”). Next, the c-ring, powered by ATP hydrolysis in V_1_, rotates clockwise through the lipid bilayer to bring the protonated c-ring glutamates close to an essential a1 residue, R740. A salt bridge forms between the protonated glutamate residue and R740, causing the release of the proton into the luminal hemichannel. Finally, the proton exits the membrane and enters the lumen by following a network of polar and negatively charged residues lining the luminal hemichannel (H748, E794, and R804), resulting in luminal acidification. For every three ATP molecules hydrolyzed, 10 protons are translocated. Amino acid positions are based on the updated accession number NM_001130021.3. The figure was created with BioRender.com.

Characterizing the structure of the V-ATPase is critical to understanding its function. The yeast V-ATPase, the most thoroughly characterized V-ATPase, has been a useful model for studying its mammalian counterpart (Toei et al., 2010). It is composed of 31 polypeptides that are organized into two distinct structural sectors: a cytoplasmic V_1_ ATPase and a membrane-embedded V_o_ proton channel (Figure 1A; Zhao et al., 2015). The yeast V_1_ region contains subunits A_3_, B_3_, C, D, E_3_, F, G_3_, and H, while the V_o_ proton channel contains subunits a, c_8_, c′, c″, d, e, f, and V_o_a1p (note: subscript number indicates number of repeating subunits) (Mazhab-Jafari et al., 2016; Roh et al., 2018). The human V-ATPase V_1_ subunit is similar to its yeast counterpart in terms of subunit type and number (Wang L. et al., 2020). However, they differ in V_o_ composition, containing the following subunits: a, c_9_, c″, d, e, Ac45 (also known as ATP6AP1 and is equivalent to yeast V_o_a1p), RNaseK (equivalent to yeast f), and ATP6AP2 (Wang L. et al., 2020). Comparing the yeast and human subunit a isoforms reveals 30–50% (up to 85%) sequence similarity (Roh et al., 2018). This high similarity increases confidence when translating yeast research to the human subunit a of interest.

### 2.1. The A_3_B_3_ hexamer

The top of the V_1_ complex is composed of an A_3_B_3_ hexamer head containing three pairs of the AB heterodimer alternatively arranged around a pseudo-6-fold symmetry axis (Figure 1A; Wang L. et al., 2020). ATP binding and hydrolysis occur between the interface of the A and B subunits (Liu et al., 1996, 1997). Initially, subunit A was believed to contain catalytic nucleotide binding sites, whereas subunit B had non-catalytic nucleotide-binding sites (Manolson et al., 1985; Feng and Forgac, 1992; Zhang et al., 1995; Vasilyeva and Forgac, 1996). The non-catalytic sites were hypothesized to be necessary to achieve maximum enzymatic activity and regulate activity in general (MacLeod et al., 1998). Contrastingly, recent evidence suggests that the mammalian V_1_ subunit does not have non-catalytic nucleotide binding sites (Abbas et al., 2020). Moreover, the A subunit in bacterial V-ATPase is thought to contain most of the catalytic residues but not all (Ueno et al., 2018). Although the specifics of the nucleotide binding site remain obscure, the ATP hydrolysis cycle within the A_3_B_3_ hexamer is well-reported. During the ATP hydrolysis cycle, the C-terminal domains of A and B undergo conformational changes depending on their bound state to ATP and ADP (Suzuki et al., 2016; Wang L. et al., 2020). First, ATP is tightly bound between the A and B subunits and is subsequently hydrolyzed into ADP and inorganic phosphate (Pi) (Figure 1B; Suzuki et al., 2016). The Pi is released and the AB pair changes conformations to a less tight ADP-bound form (Suzuki et al., 2016). This triggers the adjacent AB pair to change conformations from a semi-open (nucleotide-free) conformation to a high ATP-affinity open confirmation (Suzuki et al., 2016; Wang L. et al., 2020). Next, ATP binds tightly to the open AB pair, changing the ADP-bound dwell into a semi-open conformation, eventually releasing ADP (Suzuki et al., 2016). The conformational changes associated with the ATP hydrolysis cycle induce the tilting and oscillation of the A_3_B_3_ hexamer (Suzuki et al., 2016; Wang L. et al., 2020). These movements ultimately drive the 120^o^ rotation of the central stalk (subunits D and F), which connects the middle hole of the A_3_B_3_ hexamer to the V_o_ complex, with a torque of ∼25 pNnm (Suzuki et al., 2016; Ueno et al., 2018; Wang L. et al., 2020). The rotation of the central stalk eventually leads to the transportation of a proton across the membrane (Nishi and Forgac, 2002). Subunit A is also involved in regulating V-ATPase disassembly. The non-homologous region of subunit A, a 90-amino acid domain not present in the β-subunit of the F-ATPase, interacts with the V_o_ complex to regulate glucose-dependent V-ATPase dissociation (Shao et al., 2003; Shao and Forgac, 2004).

### 2.2. Peripheral stalks

The V_1_ and V_o_ sectors are connected by three heterodimers of E and G subunits, known as the peripheral stalks (Figure 1A; Colina-Tenorio et al., 2018). The static peripheral stalks act as the stator of a motor to counteract the torque of the central stalk (Colina-Tenorio et al., 2018). Structurally, the EG heterodimer forms a long (150 Å) right-handed coiled-coil in the middle and is stabilized at their N and C termini by forming globular domains and interacting with other subunits (Oot et al., 2012). All three peripheral stalks (EG1-EG3) share the same sequence but slightly differ in their conformations, as their N-terminal domains (NTDs), contributed by both E and G NTDs, associate with different partners in the complex (Colina-Tenorio et al., 2018; Wang L. et al., 2020). EG1 interacts with the “foot” domain of a-NTD and subunit H; EG2 links with the “head” domain of a-NTD and the “foot” domain of subunit C; and EG3 only connects to the “head” domain of subunit C (Benlekbir et al., 2012; Zhao et al., 2015; Wang L. et al., 2020). Meanwhile, the C-terminal domains (CTDs) of the peripheral stalks, contributed by both E and G CTDs, interact with the N-termini of the B subunits of the A_3_B_3_ hexamer (Benlekbir et al., 2012; Zhao et al., 2015; Wang L. et al., 2020). Along with their stator role, the peripheral stalks are thought to be crucial in the disassembly and reassembly of V-ATPases in response to extracellular stimuli (Oot et al., 2017). The regulation of V-ATPase activity through its assembly and dissociation is known as reversible dissociation. Although the exact mechanism of the peripheral stalk role in reversible dissociation remains obscure, they are permissive to the movement of the complex and the rearrangement of other subunits, which ultimately drives V-ATPase reversible dissociation (Oot et al., 2012, 2017).

### 2.3. Subunits C and H

The single subunits of the V_1_ complex, C and H, play an important role in V-ATPase reversible dissociation and overall enzymatic activity (Liu et al., 2005; Diab et al., 2009; Pérez-Sayáns et al., 2012; Oot et al., 2016; Sharma et al., 2018). Subunits C and H lie between the V_1_V_o_ interface and stabilize the peripheral stalk NTDs as aforementioned (Figure 1; Colina-Tenorio et al., 2018; Wang L. et al., 2020). Structurally, subunit C consists of three different domains: A globular ‘head’ domain made of four antiparallel β-sheets and two α-helices; a long three-helix bundle ‘neck’ domain connected by salt bridges; and a globular ‘foot’ domain composed of similar structures to the ‘head’ domain and hosts both the N and C termini (Drory et al., 2004; Wang R. et al., 2020). First identified in yeast, glucose starvation results in the dissociation of the V_1_ structure from the V_o_ sector, and reassembly is initiated upon restoration of glucose in the growth medium (Kane, 1995). Following V-ATPase dissociation, subunit C separates from both sectors and is released into the cytosol, which may be mechanistically achieved through the disruption of the interaction between subunit C and EG3 (Sumner et al., 1995; Parra and Kane, 1998; Tabke et al., 2014; Oot et al., 2017). Meanwhile, with the help of subunit H, the dissociated sectors are functionally silent as V_1_ ATPase activity is inhibited and V_o_ proton translocation is stopped (Parra et al., 2000; Couoh-Cardel et al., 2016; Oot et al., 2016). Subunit H is structurally separated into two distinct domains, connected by a flexible four-residue loop (Sagermann et al., 2001; Liu et al., 2005; Wang L. et al., 2020). The NTD, the larger of the two domains, is characterized by its repetitive arrangement of 17 right-handed α-helices (Sagermann et al., 2001). The smaller CTD is composed of eight α-helices that form two turns of a right-handed superhelix (Sagermann et al., 2001). Following the dissociation of V_1_V_o_ in yeast, the H subunit silences the enzymatic activity of V_1_, preventing erroneous ATP hydrolysis (Parra et al., 2000; Oot et al., 2016). This inhibition is initiated when the separation of the sectors causes the CTD of subunit H to move and rotate away from its binding site on a-NTD to newly bind the central rotor subunit D and the catalytic site of subunit B, trapping ADP at the catalytic site and preventing ATP hydrolysis (Parra et al., 2000; Oot et al., 2016). Contrarily, the Regulator of the H^+^-ATPase of Vacuoles and Endosomes (RAVE) complex in yeast helps in V-ATPase reassembly upon glucose readdition (Smardon et al., 2002; Smardon and Kane, 2007). The RAVE complex first associates with sector V_1_ and then binds to available subunit C (Jaskolka et al., 2021). Next, RAVE directs V_1_ and C to V_o_ and is hypothesized to accelerate their association and assembly (Jaskolka et al., 2021). In mammalian cells, rabconnectin-3α, homologous to one of the RAVE protein complexes Rav1p, is also involved in V-ATPase assembly (Einhorn et al., 2012).

### 2.4. The proteolipid c-ring

As aforementioned, the membrane-embedded V_o_ complex contains subunits a, c_9_, c″, d, e, Ac45 (also known as ATP6AP1), RNaseK, and ATP6AP2 (Wang L. et al., 2020). While the V_1_ complex is responsible for ATP hydrolysis, the V_o_ complex canonically functions as a proton channel (Figure 1; Zhao et al., 2015). A key structure of the human V_o_ complex is the c-ring, containing two proteolipid variants, c and c″, in a 9:1 ratio (Figure 1A; Abbas et al., 2020; Wang L. et al., 2020). The c and c″ subunits are highly hydrophobic and are composed of four and five transmembrane helices, respectively (Flannery et al., 2004). Functionally, the c-ring is primarily involved in proton translocation, which is mediated by a key glutamic acid residue buried in each c/c″ subunit, undergoing reversible protonation (Figures 1A,B; Hirata et al., 1997). The conserved glutamic acid residues are E139 on subunit c and E98 on subunit c″, according to UniProt (P27449, Q99437) (Wang L. et al., 2020). To achieve proton translocation, the c-ring must rotate clockwise relative to the adjacent stator subunit a (Forgac, 2007). This movement is powered by ATP hydrolysis in V_1_, rotating the central stalk, which is linked to the c-ring via subunit d (Figure 1B; Iwata et al., 2004; Wang L. et al., 2020). The cone-shaped subunit d sits on top of the c-ring and attaches to the central stalk on its concave surface in a shape-complementary manner (Wang L. et al., 2020). The convex or N-terminus side of subunit d interacts with the c-ring, specifically at the cytosolic N-terminus loop of c″ (Roh et al., 2018; Wang L. et al., 2020). The d-to-c-ring interaction is reinforced by residues forming hydrogen bonds, hydrophobic contacts, and a salt bridge (Roh et al., 2018; Wang L. et al., 2020). Secondarily to proton transportation, the c-ring has a role in membrane fusion and neurotransmission, acting as a large-conductance transmembrane protein pore (Couoh-Cardel et al., 2016). This coincides with the knowledge that the V_o_ complex is implicated in exocytosis (Maxson and Grinstein, 2014; Morel and Poëa-Guyon, 2015).

### 2.5. V-ATPase subunit a

**Figure 2:**
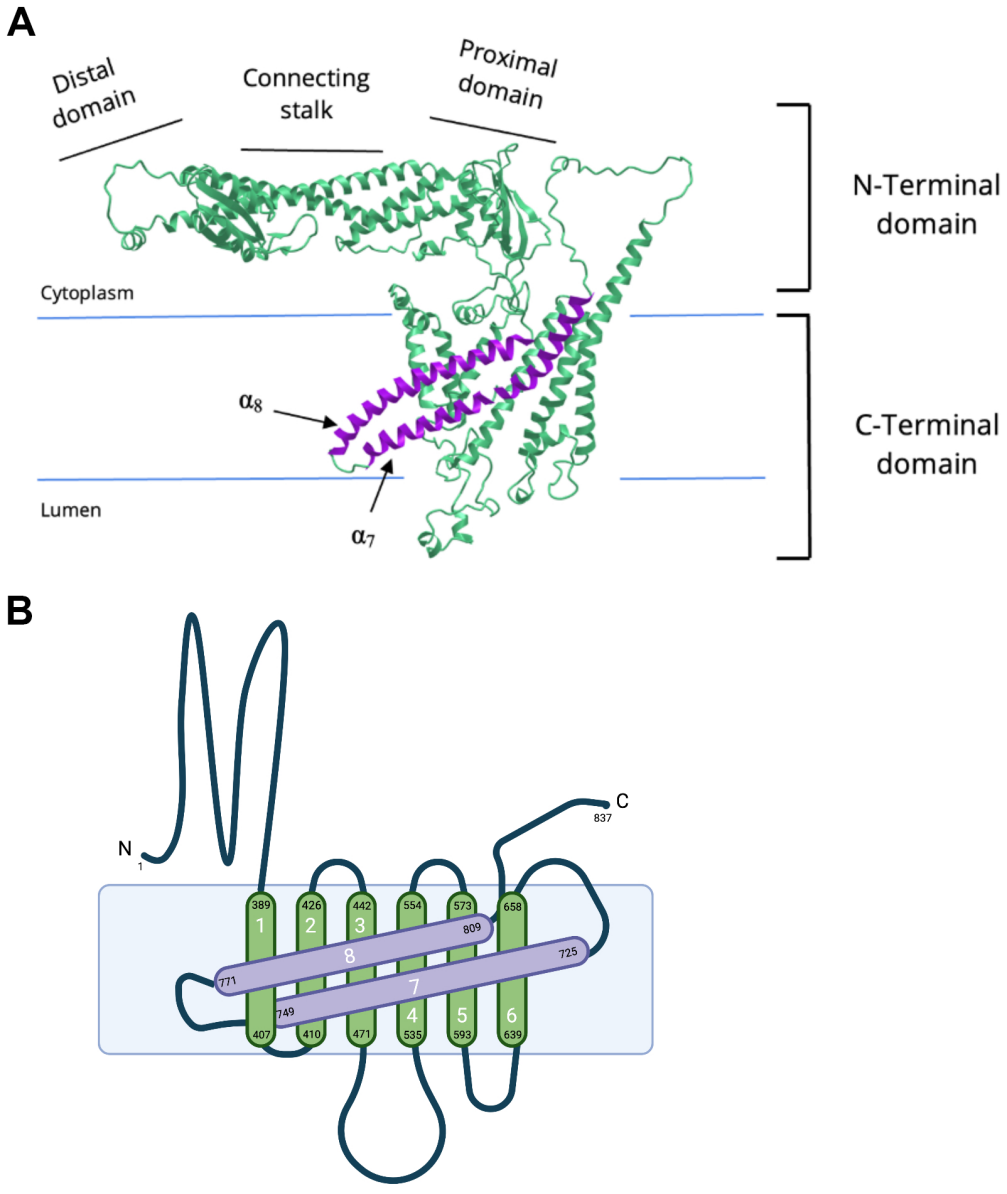
Cryo-EM of NTD and CTD of subunit a1. **(A)** Cryo-EM derived structure of the human V-ATPase subunit a (RCSB PDB: 6WLW, source: iCN3D) (Wang J. et al., 2020; Wang L. et al., 2020) and **(B)** a simplified topology representation of V-ATPase subunit a1. Subunit a1 is made up of two domains, a cytoplasmic NTD and membrane-embedded CTD. The a1-NTD is composed of a distal domain, connecting stalk, and proximal domain. a1-CTD consists of eight transmembrane α-helices connected by short linker loops on both cytoplasmic and luminal sides. a1-CTD is important for proton translocation. Transmembrane helices α_7_-α_8_ (in purple) are tilted. These transmembrane helices compose part of the hemichannels and hold important residues for proton translocation (R740 at α_7_ and R804 at α_8_). **(B)** was created with BioRender.com.

Subunit a of the V_o_ complex plays a crucial role in coupling ATP hydrolysis and proton translocation. Subunit a consists of a cytosolic N-terminal domain and a membrane-integrated C-terminal domain (Figure 2A) with eight transmembrane 𝛼-helices (Figure 2B). The highly tilted 𝛼7 and 𝛼8 are in contact with the c-ring (Figure 2B), forming the two offset hemichannels responsible for proton translocations (Mazhab-Jafari et al., 2016; Abbas et al., 2020). In yeast, there are two known isoforms of subunit a: the Vph1p and Stv1p, while there are four in fruit flies, worms, mice, and humans: a1, a2, a3, and a4 (Manolson et al., 1994; Nishi and Forgac, 2000; Toyomura et al., 2000; Oka et al., 2001a,b; Wagner et al., 2004). Each isoform is enriched in a specific subcellular location, making subunit a the determining factor for V-ATPase proper targeting. A chimeric study showed that the targeting information is controlled by the cytosolic N-terminal domain of subunit a in yeast and that Vph1p is localized at the vacuole, while Stv1p is at the Golgi (Kawasaki-Nishi et al., 2001b). Stv1p’s sorting signal on W^83^KY residues was found through a random mutagenesis study (Finnigan et al., 2012). However, unlike yeast, the specific targeting signal of mammalian subunit a remains elusive.

Subunit a1 is the neural isoform. In fruit flies, it is located in synaptic vesicles and membranes of presynaptic nerve terminals, where it interacts with Ca^2+^-Calmodulin in regulating SNARE assembly, which is important for Ca^2+^-dependent spontaneous neurotransmitter release (Zhang et al., 2008; Wang et al., 2014). Meanwhile, isoforms a2 and a3 are ubiquitously expressed with a2 located in the Golgi and a3 targeted to endosomes and lysosomes. Isoforms a3 and a4 are also localized to plasma membranes, with a3 found in developing osteoclasts and a4 in ion-transporting epithelia of the kidneys, ocular ciliary bodies, human ears, and epididymal cells (Oka et al., 2001a; Stover et al., 2002; Kawamura et al., 2010).

#### 2.5.1. CTD of subunit a1

Subunit a1 is organized into two distinct domains, the cytoplasmic N-terminal domain (residues 1–388) and the membrane-embedded C-terminal domain (residues 389–837), each consisting of ~400 residues (Figures 1A, 2; Roh et al., 2018). The CTD of a1 is involved in proton translocation and includes eight transmembrane α-helices, with additional short linker-loops at the cytoplasmic and luminal sides (Figure 2; Roh et al., 2018; Abbas et al., 2020). Overall, the membrane-embedded a1 CTD is arranged beginning with the short, cytoplasmic-facing helices α_1_ and α_2_ [CTD α-helix numbering according to Toei et al. (2011)] (Mazhab-Jafari et al., 2016; Abbas et al., 2020). These two α-helices partially cross the lipid bilayer and are slightly exposed to the cytoplasm (Mazhab-Jafari et al., 2016; Abbas et al., 2020). Transmembrane helices α_3_-α_6_ form a central layer and are subsequently followed by the long and heavily tilted α_7_ and α_8_ helices (Figure 2A), terminating the CTD and forming most of the interface between subunit a1 and the c-ring, along with transmembrane helices of c_1_ and c″ (Mazhab-Jafari et al., 2016; Roh et al., 2018; Abbas et al., 2020). Moreover, the a1-CTD forms two aqueous cavities that extend only part way through the membrane and interface the c-ring, known as hemichannels (Mazhab-Jafari et al., 2016; Roh et al., 2018; Abbas et al., 2020). One cavity faces the cytoplasmic side, whereas the other faces the lumen, providing access to proton entry and exit for (from) the conserved glutamic acid residues on the c-ring (Figures 1A, C; Kawasaki-Nishi et al., 2001b; Mazhab-Jafari et al., 2016; Roh et al., 2018; Abbas et al., 2020). The cytoplasmic cavity is made from the cytoplasmic ends of α-helices 4, 5, 7, and 8, lined with charged and polar residues (Roh et al., 2018). The luminal-facing cavity is composed of the loops connecting α-helices 3, 4, 7, and 8, lined with several polar residues (Roh et al., 2018). In yeast, the key polar residues in the cytoplasmic cavity are E721, N725, and H729, which one is conserved in humans as E726 according to UniProt sequence alignment (P32563, Q93050) (Roh et al., 2018; Vasanthakumar and Rubinstein, 2020). The important yeast residues in the luminal cavity, D425, D481, and H743 correspond as D409, D466, and H748 in humans, according to UniProt sequence alignment (P32563, Q93050) (Roh et al., 2018; Vasanthakumar and Rubinstein, 2020). Non-conservative mutations to these residues lead to a significant loss in proton translocation activity, highlighting their importance (Toei et al., 2011). Furthermore, a single glycosylation at residue N488 of the first a1 luminal loop is required for protein stability and the incorporation of subunit a1 into the V-ATPase itself (Esmail et al., 2018b).

#### 2.5.2. Subunit a1 and the proton translocation pathway

Mammalian proton transport requires collaboration between subunit a1 and the c-ring. Although there is little information regarding the proton transport system for mammalian V-ATPase, researchers have uncovered key residues and interactions within the proton transport pathway in yeast (Kawasaki-Nishi et al., 2003; Toei et al., 2011; Wang et al., 2014; Mazhab-Jafari et al., 2016; Roh et al., 2018; Abbas et al., 2020). Proton transport begins with the protonation of the essential glutamic acid residues on the c-ring (E139 subunit c and E98 subunit c” in humans; E137 subunit c, E145 subunit c’, and E108 subunit c” in yeast) (Figures 1A, C; Kawasaki-Nishi et al., 2001b; Roh et al., 2018; Abbas et al., 2020). Protonation of the c-ring glutamic acid residues occurs in the cytoplasmic cavity of subunit a, mediated by α_7_ residues S728 and E721 (conserved as S733 and E726 in human subunit a1) (Roh et al., 2018). The now neutrally charged c-ring glutamate residues are stabilized by hydrogen bonds formed with neighboring c-ring tyrosines, enabling their stable entry into the lipid bilayer (Roh et al., 2018; Abbas et al., 2020). Next, the c-ring, powered by ATP hydrolysis in V_1_, rotates clockwise through the lipid bilayer to bring the protonated c-ring glutamates close to an essential residue, R735, located on α_7_ (conserved as R740 in human subunit a1) (Wang et al., 2004; Abbas et al., 2020). A salt bridge forms between the protonated glutamate residue and R735/R740, causing the release of the proton into the luminal cavity (Roh et al., 2018; Abbas et al., 2020). Finally, the proton exits the membrane and enters the lumen by following a network of polar and negatively charged α_7-8_ residues lining the luminal cavity (Abbas et al., 2020). These charges residues include H743, E789, and R799 in yeast, which are conserved as H748, E794, and R804 in the human subunit a1, according to UniProt sequence alignment (P32563, Q93050) (Forgac, 2007; Abbas et al., 2020; Vasanthakumar and Rubinstein, 2020). For every three ATP molecules hydrolyzed, 10 protons are translocated (Abbas et al., 2020).

Alternatively, Roh et al. (2018) hypothesize that the deprotonation of the c-ring glutamates occurs at E789 in yeast via hydrogen bonding to neighboring proton binding site tyrosine residues. E789 then transfers the proton into the lumen using a network of amino acids, including D425, D481, and H743 (Roh et al., 2018). The deprotonated glutamate residues then interact with S792 and H796 before forming a salt bridge R735, acting as a fail-safety mechanism to ensure the deprotonation of the glutamates near the luminal cavity.

#### 2.5.3. NTD of subunit a1

The NTD of subunit a1 forms a hairpin-like structure consisting of two globular segments, the proximal and distal domain, connected by a long coiled-coil known as the connecting stalk (Figure 2; Srinivasan et al., 2011; Abbas et al., 2020; Wang L. et al., 2020). In yeast, different subunit a isoforms have preferential organelle targeting, which is mediated by the a-NTD (Kawasaki-Nishi et al., 2001b). The N-terminus of the Vph1 isoform directly interacts with the phosphatidylinositol phosphate lipid PI(3,5)P2 for its recruitment to vacuoles (Kawasaki-Nishi et al., 2001b; Banerjee et al., 2019). Likewise, the Stv1 isoform is localized to the Golgi via its interaction with PI(4)P (Kawasaki-Nishi et al., 2001b; Banerjee and Kane, 2017). However, the specific cell-targeting information of the mammalian homologue remains unclear. As aforementioned, EG1 interacts with the “foot” (distal) domain of subunit a1, serving as a stator to anchor EG1 (Figure 1A; Qi and Forgac, 2008; Colina-Tenorio et al., 2018). This interaction occurs through the NTDs of subunits E and G that compose EG1, along with location-specific residues of the proximal a-NTD portion (residues 347–369 in yeast, 331–385 in human a1) (Qi and Forgac, 2008). Conformational changes to this interaction are also hypothesized to trigger the dissociation of the V_o_ and V_1_ sectors (Qi and Forgac, 2008). Specifically, this interaction, along with contact from the C-terminal helices of subunit H, may create an environment that keeps subunit a1 in a conformation favorable for proton translocation (Wang R. et al., 2020). Furthermore, both the proximal and distal domains of a1-NTD bind to subunit d when V_o_ is separated from V_1_ (Qi and Forgac, 2008; Roh et al., 2018). This interaction locks the rotary subunit, preventing rotation and proton translocation during V_o_ dissociation (Nishi and Forgac, 2000; Roh et al., 2018).

## 3. Subunit a1-associated neurological disorders

The V-ATPase plays a crucial role in maintaining the homeostatic pH of intracellular vesicles and mutations that impair its function are implicated in various diseases. As the V_o_ complex couples ATP hydrolysis with proton translocation, many variants to genes encoding subunit a1 have debilitating effects on vesicle acidification, which affects lysosomal activities and neurotransmission.

### 3.1. Variants of *ATP6V0A1* in developmental and epileptic encephalopathies (DEE) and progressive myoclonus epilepsy (PME)

Recently, two groups of researchers identified *de novo* and biallelic variants of *ATP6V0A1*, encoding the a1 subunit, in individuals with rare neurological disorders: DEE and early onset PME (Aoto et al., 2021; Bott et al., 2021). DEE is a condition consisting of both severe epilepsies and significant cognitive developmental delay or loss (encephalopathies), which manifest early in life. Increasing evidence has attributed DEE to various genetic causes, which are believed to be responsible for epilepsy and adverse encephalopathy independently (Berg et al., 2021). PME is a group of rare disorders with symptoms including myoclonus (sudden muscle jerking), epilepsy, and progressive neurological deterioration. While also uncommon, the onset of this disease is more variable than DEE, ranging from early childhood to adolescence and adulthood (Orsini et al., 2019).

**Figure 3:**
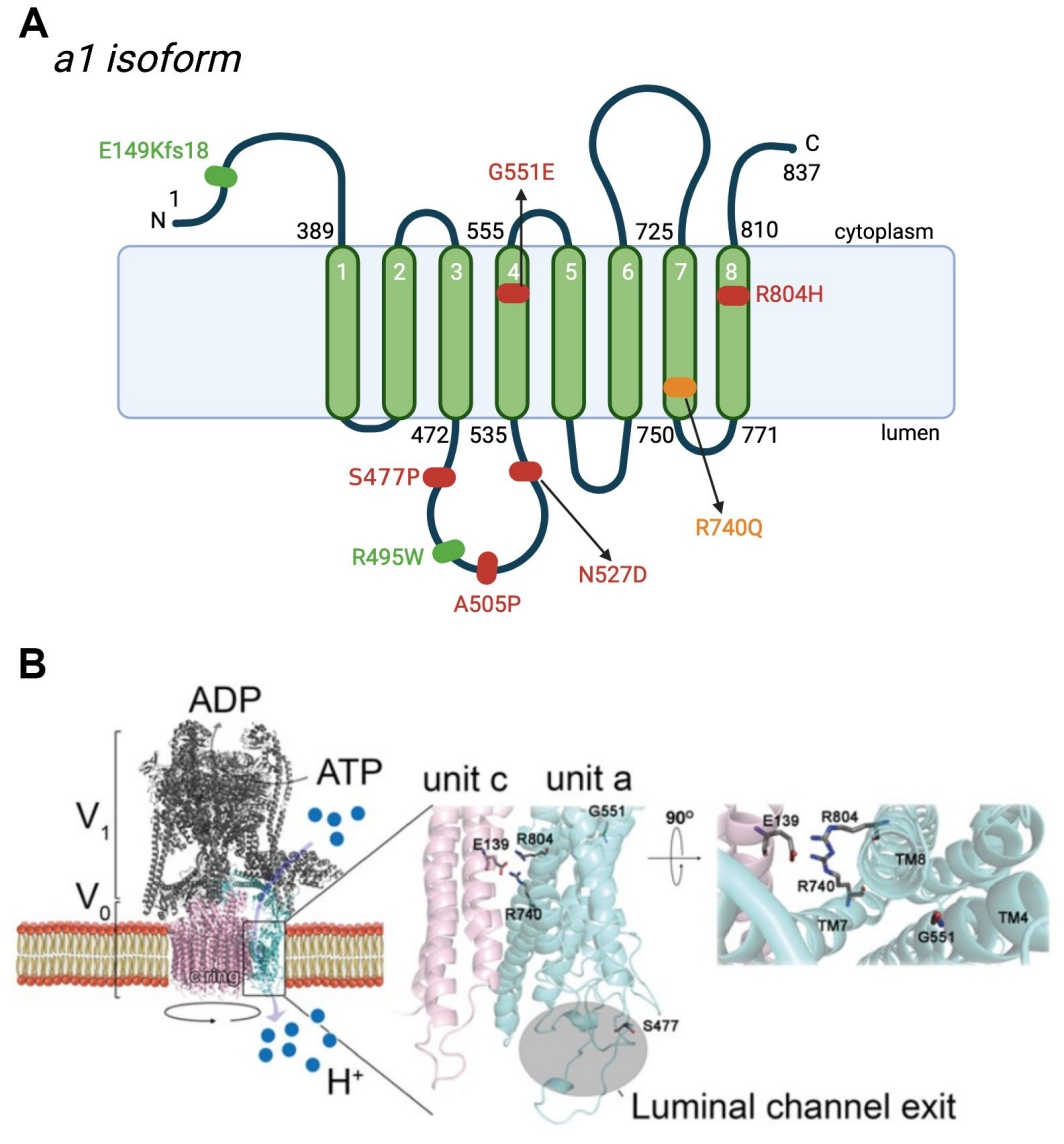
*De novo* and biallelic mutations to *ATP6V0A1* impair V-ATPase functions in cases of DEE and PME. **(A)** Schematic representation of the location of mutations to subunit a1 in DEE (red), early onset PME (green), and both diseases (yellow) (Aoto et al., 2021; Bott et al., 2021). The most common mutation is R740Q, which is *de novo*, heterozygous, and follows a dominant inheritance pattern. Other *de novo* heterozygote variants are S477P, G551E, and R804H. Biallelic mutations, E149Kfs18 and R495W, are reported in 5 individuals with PME, 4 of whom are from the same family. In DEE individuals, A505P and N527D are in compound heterozygosity, each with a 50-kb deletion [del(17)(q21.2)] and a splice site mutation (c.196C + 1G > A), respectively. Positions are based on the updated accession number NM_001130021.3. For the purpose of annotation, transmembrane α_7_-α_8_ are not tilted. Amino acid sequences are based on Q93050 in UniProt. **(A)** was created with BioRender.com. **(B)** Homology model of human V-ATPase and locations of residues reported in *de novo* mutations on subunit a1 (Bott et al., 2021). Residues S477, G551, R740, and R804 are represented as stick structures relative to the key glutamic acid residue, E139 of subunit c. S477, at the second short linker loop (connecting transmembrane α_3_-α_4_) of luminal hemichannel.

**Figure 4:**
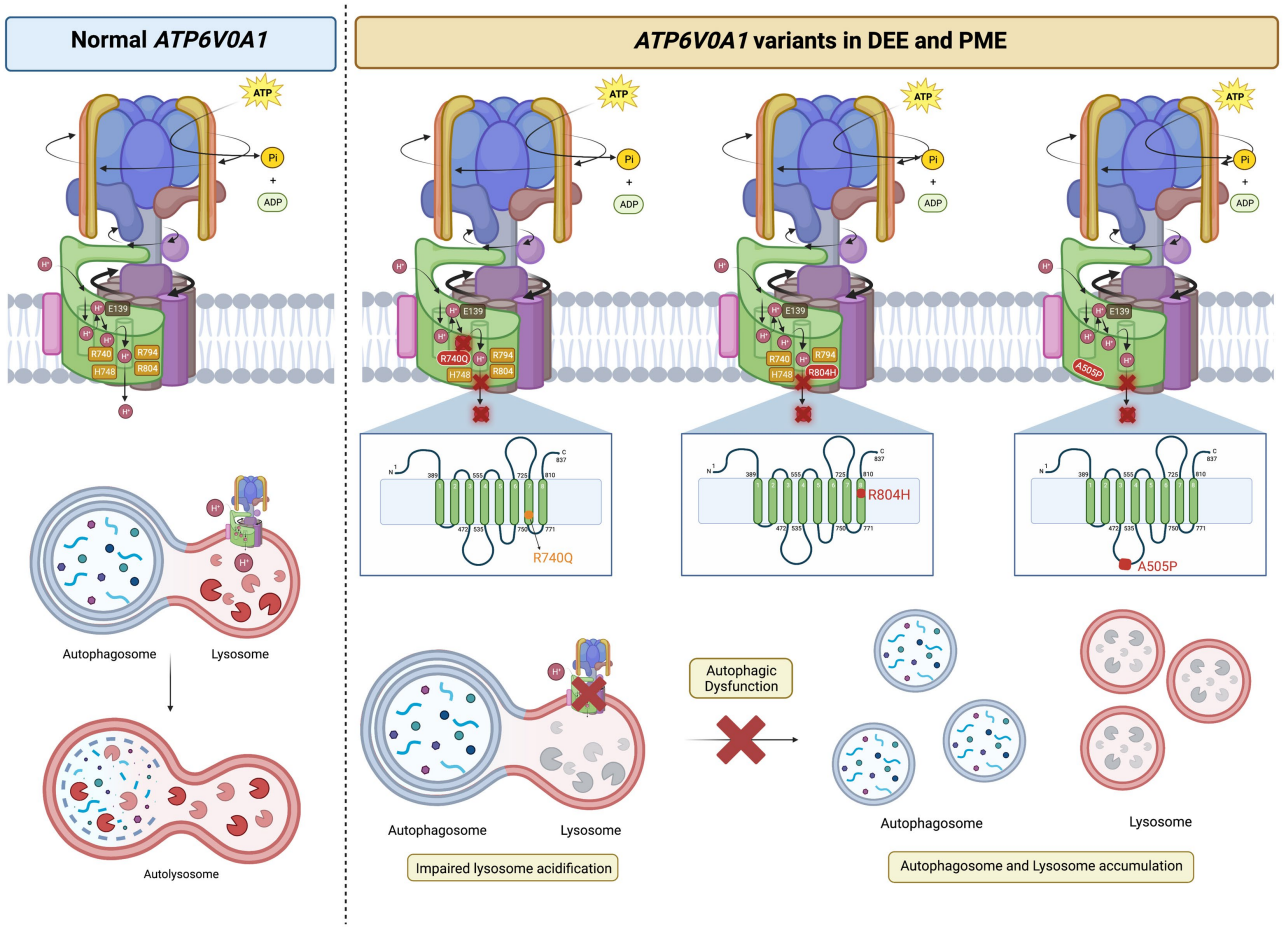
*ATP6V0A1* variants implicated in DEE and PME cause lysosomal and autophagic dysfunction. Normal *ATP6V0A1* encodes for a functional subunit a1 of the V-ATPase, which couples ATP hydrolysis and proton translocation through the c-ring. Protons are pumped into the lumen of lysosomes to achieve a pH of ~5.5 for optimal enzymatic activities in autophagosome clearance. On the other hand, variants of *ATP6V0A1* compromise V-ATPase function and interfere with proper lysosomal acidification, leading to the accumulation of autophagosome and lysosomal bodies, as reported in cases of DEE and PME. R740Q mutant was reported in DEE and PME patients, while R804 and A505P were found in DEE patients (Aoto et al., 2021; Bott et al., 2021). Based on studies in animal models by Aoto et al. (2021) and Bott et al. (2021), R740Q and R804H in the 7^th^ and 8^th^ transmembrane region, respectively, are key residues in the proton translocation, adversely affected by such substitutions. A505P variant on the 2^nd^ luminal linker region is also implicated in DEE, affecting autophagic function (Aoto et al., 2021). It is still unclear how A505P impairs V-ATPase function in autophagic dysfunction. The figure was created with BioRender.com.

The first group, Aoto et al. (2021) studied individuals with DEE and identified two unrelated individuals with the same *de novo* heterozygous missense mutation, R741Q (corresponds to R740Q (c.2219G > A), based on the updated accession number NM_001130021.3) (Figures 3A, 4; Table 1). Additionally, they identified two inherited missense mutations, A512P and N534D, each in compound heterozygosity with a 50-kb deletion (c.del(17)(q21.2)) and a splice site mutation (c.196 + 1G > A), respectively. According to the updated accession NM_001130021.3, the aforementioned mutations are A505P (c.1513C > G) and N527D (c.1579A > G) (Figures 3A, 4; Table 1). Using an *in vitro* culture, authors demonstrated that when homozygous, all missense mutations mentioned above resulted in a loss of function and higher lysosomal pH. However, upon further investigation using a mouse model, Aoto et al. (2021) showed that they do so at different degrees. They generated mice harboring human A505P and R740Q variants, corresponding to A506P and R741Q in mice (note: mice lines are named based on human residue variants). *Atp6v0a1^R740Q/R740Q^* embryonic lethality suggests this mutation leads to a complete V-ATPase loss-of-function which impairs neuronal development. On the other hand, *Atp6v0a1^A505P/A505P^* survived for 2 weeks and displayed impaired motor function and ataxia. Nonetheless, these pups had smaller brains and fewer neurons, suggesting that the A505P mutation affects neurodevelopment and synaptic formation. This retardation in cell growth is due to A505P effect on mTORC1, a signaling pathway involved in the regulation of cell proliferation and differentiation. Immunoblot analysis showed a decrease in the phosphorylated S6 level, which is a downstream target of mTORC1. Additionally, *Atp6v0a1^A505P/A505P^* affects the expression of other V-ATPase subunits, as shown by a decrease in the A subunit of the V_1_ complex in the cerebellum (encoded by *ATP6V1A*).

**Table 1:** List of *ATP6V0A1, ATP6V0A2, ATP6V0A3*, and *ATP6V0A4* variants.

Gene	Nucleotide change	Amino acid change	Mutation type	Zygosity	Impact on V-ATPase	Associated disease	Variant identifier	Reference
*ATP6V0A1*	c.T1429C	p.S477P	Missense	Heterozygous	Alters conformation of luminal channel, hindering proton translocation	DEE	VAR_087489	Bott et al. (2021)
*ATP6V0A1*	c.C1483T	p.R495W	Missense	Compound heterozygous with frameshift mutation: c.445delG, p.E149Kfs18	Loss of positively charged residue near the luminal channel exit hinders proton translocation	PME and ataxia	rs781278654	Bott et al. (2021)
*ATP6V0A1*	c.C1513G	p.A505P	Missense	Compound heterozygous with deletion: c.del(17)(q21.2)	Undetermined	DEE	VAR_087491	Aoto et al. (2021)
*ATP6V0A1*	c.A1579G	p.N527D	Missense	Compound heterozygous with splice site mutation: c.196+1G>A	Undetermined	DEE	rs766856192	Aoto et al. (2021)
*ATP6V0A1*	c.G1652A	p.G551E	Missense	Heterozygous	Deforms environment mediating proton exchange	DEE	VAR_087493	Bott et al. (2021)
*ATP6V0A1*	c.G2219A	p.R740Q	Missense	Heterozygous	Impedes glutamate protonation thereby disrupting proton translocation	DEE, PME, autism, microcephaly	rs1567871600	Aoto et al. (2021); Bott et al. (2021)
*ATP6V0A1*	c.G2411A	p.R804H	Missense	Heterozygous	Impedes glutamate protonation thereby disrupting proton translocation	DEE	VAR_087495	Bott et al. (2021)
*ATP6V0A2*	c.187T	p.R63X	Nonsense	Homozygous	Truncated protein, non-functional subunit a2	ARCL II	rs80356750	Kornak et al. (2008)
*ATP6V0A2*	c.C1214T	p.P405L	Missense	Homozygous	Compromises subunit a2 stability, complex assembly and Golgi trafficking	ARCL II	rs750788949	Hucthagowder et al. (2009); Esmail et al. (2018a)
*ATP6V0A2*	c.C2293T	p.Q765X	Nonsense	Homozygous	Truncated protein, non-functional subunit a2	ARCL II	rs80356758	Kornak et al. (2008)
*ATP6V0A2*	c.C2432C	p.L811P	Missense	Compound heterozygous with splicing mutation: c.2055+2dupT	Changes last α-helix conformation, hindering subunit interactions and proton translocation	ARCL II	rs778642108	Fischer et al. (2012)
*ATP6V0A3*	c.IVS2+4A→T	p.V26-D39del	Deletion	Homozygous	Unable to assemble with V1 subunit	MIOP	N/A	Kornak et al. (2000)
*ATP6V0A3*	c.G1024T	p.E342X	Stop	Heterozygous	Truncated protein, non-functional subunit a3	MIOP	N/A	Sobacchi et al. (2001); Susani et al. (2004)
*ATP6V0A3*	c.G1213A	p.G405R	Missense	Homozygous or compound heterozygous with R444L	Steric hindrance of 1st cytoplasmic loop	MIOP	VAR_019569	Sobacchi et al. (2001); Susani et al. (2004)
*ATP6V0A3*	c.G1331T	p.R444L	Missense	Homozygous or compound heterozygous with G405R	Protein instability due to hydrophobic Arginine (R)	MIOP	rs137853151	Sobacchi et al. (2001); Susani et al. (2004)
*ATP6V0A3*	c.G2412A	p.W805X (updated NM_006019.4)	Stop	Heterozygous	Truncated protein, non-functional subunit a3	MIOP	rs1555000308	Kornak et al. (2000)
*1. ATP6V0A4 2. ATP6V1B1*	1.c.C419T 2.c.A437G	1. p.T140M 2. p.D146G	Missense	Digenic heterozygous	1. Slight conformational change hindering subunit trafficking and assembly 2. Alteration of subunit surface charge leading to the disruption of ATP binding sites	dRTA	1. rs144802156 2. rs782681801	Nagara et al. (2018)
*ATP6V0A4*	c.C1631T	p.S544L	Missense	Heterozygous	Unable to bind to B1 subunit, assembly issues, weakened ATPase activity	Complete and incomplete dRTA	rs1026435	Imai et al. (2016); Chen et al. (2020)
*ATP6V0A4*	c.G2420A	p.R807Q	Missense	Homozygous	Reduction of subunit a4 production leading to V-ATPase assembly and activity deficits	dRTA, Sensorineural hearing loss	rs28939081	Stover et al. (2002)
*ATP6V0A4*	c.G2458C	p.G820R	Missense	Homozygous	Loss of a4 binding to phosphofructokinase-1, formation of salt bridge disrupting proton translocation	dRTA	rs267606671	Esmail et al. (2018a)

ARCL II, autosomal recessive cutis laxa type II; DEE, developmental and epileptic encephalopathies; dRTA, distal renal tubular acidosis; MIOP, malignant infantile osteopetrosis; PME, progressive myoclonus epilepsy.

Staining of *Atp6v0a1^A505P/A505P^* brain sections revealed lower levels of mature Cathepsin D, abnormal distribution of lysosomes, and an increase in neuronal cell death (Aoto et al., 2021). Cathepsin D is an aspartic protease whose trafficking and maturation in the endolysosomal compartment depends on optimum acidic pH (pH 3–5) (Steinfeld et al., 2006). This suggests that *Atp6v0a1^A505P/A505P^* impairs lysosomal activity, leading to an abnormal cellular distribution of lysosomes and consequently, cell death. Accordingly, the authors attributed the decrease in mTORC1 signaling and increased neuronal cell death induced by lysosomal dysfunction to the *Atp6v0a1^A505P/A505P^* pup’s smaller brain size. Furthermore, lysosomal dysfunction leads to failure in autophagosome clearance, as shown by the accumulation of autophagosomes and lysosomes in *Atp6v0a1^A505P/A505P^*. Additionally, *Atp6v0a1^A505P/A505P^* electrophysiology analysis reported lower amplitude and frequency of miniature excitatory postsynaptic current (mEPSC) and miniature inhibitory postsynaptic current (mIPSC) relative to the wildtype group. Thus, it suggests a reduction in neurotransmitter content inside synaptic vesicles (reduced neurotransmitter loading) and a decrease in vesicle fusion events for release. Therefore, the V-ATPase plays a crucial role in determining synaptic vesicle content and release.

The Aoto et al. (2021) *Atp6v0a1*^A505P/A505P^ mouse model allows for a deeper investigation of *ATP6V0A1* missense mutations and their impact on a1 subunit synthesis, protein complex assembly, V-ATPase loss of function, along with their effects on lysosome and autophagosome function, and mTORC-mediated neuronal development. Additionally, this study showed the importance of the a1 linker loop in proton translocation. Thus, the mouse model warrants further investigation on *ATP6V0A1* variants with mutations on linker loops (not only on the transmembrane α-helices), such as S477P, G551E, and R495W, and how such mutations lead to impaired proton translocation.

The second group, Bott et al. (2021) identified 12 individuals with *de novo* missense variants to *ATP6V0A1* presenting phenotypes of DEE and five individuals with biallelic variants showing early onset PME with ataxia. R740Q is the most common *de novo* missense variant, found in 8 out of 12 individuals with DEE. Identification of this mutation hotspot in multiple unrelated DEE individuals provides stronger evidence of the causal relation between this candidate *de novo* mutation with DEE. They also identified three other *de novo* heterozygous variants: S477P (c.1429 T > C), G551E (c.1652G > A), and R804H (c.2411G > A) on the third linker loop domain (between transmembrane α-helix 3 and 4), α_4_, and α_8_, respectively, (Figure 3A). In five patients with early onset PME with ataxia (four are from the same family), they found a novel compound heterozygous *ATP6V0A1* variant: E149Kfs18 (c.445delG) and R495W (c.1483C > T) (Figure 3A; Table 1).

To circumvent the embryonic lethality in the mouse model reported by Aoto et al. (2021), Bott et al. (2021) conducted a R740Q *in vitro* cell culture study, which revealed impaired protonation, abolished level of Cathepsin D, and accumulation of LC3-II, a standard marker for autophagosomes. These results indicate impaired lysosome activity and autophagosome turnover. Next, Bott et al. (2021) observed *Caenorhabditis elegans* harboring the *unc-32* mutation, an *ATP6V0A1* ortholog. They found that knockdown of *unc-32* increases endogenous LGG-1::mCherry fluorescence (nematode ortholog of LC3) without increasing its expression level, suggesting impaired protein clearance. *unc-32* knockdown reduced the expression of autophagic machinery components and lysosomal hydrolytic enzymes while increasing *sqst-1* expression, a stress-responsive autophagic receptor. Moreover, *C. elegans* with the corresponding R740Q homozygous mutation, *unc-32(rm20)*, showed developmental arrest and the accumulation of proteins in the nerve ring.

Homology models in yeast and fly demonstrate the importance of residue R740 for proton translocation. A yeast study showed the importance of residue R735 of Vph1p for proton translocation (R740 in human *ATP6V0A1*) (Kawasaki-Nishi et al., 2001a). R735K leads to a complete loss in proton transport while retaining approximately 20% of the wild-type ATP hydrolysis function. On the other hand, R735 substitution with Asn, Glu, or Gln results in the complete impairment of ATP hydrolysis and proton transport function. This suggests that any substitution, including conserved substitutions (ex. R735K), cannot rescue enzymatic function, highlighting the importance of this residue. Moreover, *Drosophila melanogaster* harbouring a homologous mutation (R755A in *VHA100-1*) showed lysosomal abnormality and autophagosome accumulation (Williamson et al., 2010).

By forming a salt bridge, R740 removes a proton bound to a glutamic acid residue of the proteolipid ring subunit c (E139), allowing its translocation into the lumen (Figures 1C, 3B; Aoto et al., 2021; Bott et al., 2021). The missense mutations, R740Q and R804H, impair this crucial deprotonation process (Figures 3B, 4). A more extensive understanding into the role of R740 in proton translocation requires conducting studies using a conditional or inducible knockout mice model. These models could potentially circumvent embryonic lethality and will help explain the pathophysiology of R740Q variant-related diseases in the mammalian system. Additionally, future *in vitro* and *in vivo* analysis of the R804H is important to complete our understanding of proton translocation throughout the luminal hemichannel and how R804 mutations translate clinically. Moreover, the G551A variant on α_4_ alters the structure of the protein region crucial for proton exchange (Figure 3). The luminal domain variant, S477P, modifies the loop contour of the luminal hemichannel, impairing proton translocation (Figure 3B). Altogether, these findings emphasized the importance of the a1 subunit for proton translocation, especially residue R740, identified in most cases of DEE and PME with *ATP6V0A1* mutations.

Independent of its role in acidification, the V_o_ complex is suggested to be required for membrane fusion (Peters et al., 2001; Hiesinger et al., 2005; Peri and Nüsslein-Volhard, 2008; Saw et al., 2011; Wang et al., 2014). The direct interaction between subunit a1/V100 (subunit a1 *Drosophila* ortholog) and soluble N-ethylmaleimide-sensitive factor activating protein receptors (SNAREs) has also been well documented (Perin et al., 1991; Galli et al., 1996). SNARE proteins mediate vesicle fusion by tethering vesicles to their target membrane (Söllner et al., 1993). A V100 loss-of-function study showed that V100 regulates synaptic vesicle fusion downstream of SNARE-dependent vesicle priming (Hiesinger et al., 2005). Moreover, in mouse hippocampal neurons, subunit a1 is not directly involved in vesicle fusion but instead modulates neurotransmitter release upstream of docking, favoring the fusion of acidified and loaded synaptic vesicles (Bodzęta et al., 2017). Wang et al. (2014) also proposed an acidification-independent mechanism whereby a1/V100 interacts with SNAREs to mediate SNARE assembly and spontaneous release, in a Ca^2+^–Calmodulin -dependent manner. Without Ca^2+^–Calmodulin, V100 competitively binds to the target-membrane SNAREs synaptobrevin and syntaxin, disrupting SNARE complex formation and hindering spontaneous release. However, the addition of Ca^2+^–Calmodulin rescues SNARE assembly. This process is mediated by the NTD of V100, as it hosts key SNARE and Calmodulin binding residues (Hiesinger et al., 2005; Zhang et al., 2008; Wang et al., 2014). Furthermore, this study provides evidence that subunit a1 is involved in the regulation of exocytosis, but does not contribute to the fusion pore, unlike the c-ring and other V_o_ subunits (Couoh-Cardel et al., 2016).

Additional findings on the reduction in spontaneous neurotransmitter release (mEPSC and mIPSC) in the A505P variant, reported by Aoto et al. (2021), highlights the critical role of V_o_a1-containing V-ATPase in driving neurotransmitter uptake and overall neurotransmission. V_o_a1 and V_o_a2 are the two isoforms found on secretory vesicles and contain critical and overlapping roles in acidification and neurotransmitter uptake (Kawasaki-Nishi et al., 2001b). Loading neurotransmitters into synaptic vesicles requires the collaborative work of a V-ATPase and a vesicular neurotransmitter transporter. The V-ATPase creates an electrochemical gradient which is utilized by the vesicular neurotransmitter transporter to pump neurotransmitters into the vesicular lumen. Recently reported, ATP concentration regulates V-ATPase pumping probability, while the electrochemical proton gradient controls the pumping rate (Kosmidis et al., 2022). Investigations on V_o_a1-containing V-ATPase role in neurotransmitter loading have been difficult since knockout models of this key subunit are lethal (Dickinson et al., 2016; Ma et al., 2019). Thus, the *Atp6v0a1^A505P/A505P^* mouse model creates an opportunity to further examine the effects of V_o_a1 mutations on V-ATPase proton pumping kinetics and neurotransmitter loading, to better comprehend its role in secretory vesicles and related pathophysiology, including DEE and PME-related seizures resulting from excitatory and inhibitory imbalance.

### 3.2. Impaired subunit a1 trafficking due to mutations in presenilin-1 and Alzheimer’s disease (AD)

In addition to impaired V-ATPase function due to mutations within the a1 subunit itself, studies have reported variants in V_o_a1-interacting proteins affecting the a1 activity. Namely, mutations in presenilin-1 (PS-1) which is implicated in AD. AD is the most common cause of dementia and is characterized by impaired memory and cognitive abilities. AD neuropathology has been associated with two types of protein depositions: initiation by extracellular Amyloid-β (Aβ) accumulation, followed by hyperphosphorylation of intracellular tau protein forming the neurofibrillary tangle. Although studies have also reported tau pathology progression independently of Aβ (van der Kant et al., 2020). Aβ originates from the cleavage of an Amyloid Precursor Protein (APP) by extracellular β- and intracellular 𝛾-secretases. PS-1 is a component of the 𝛾-secretase responsible for the intracellular cleavage and processing of APP. Most familial forms of AD (FAD) are associated with mutations to PS-1, resulting in an increased formation of Aβ aggregates (Lee et al., 2010). Besides promoting the accumulation of Aβ plaque, loss-of-function of PS-1 affects the proper targeting of V_o_a1-containing V-ATPases to lysosomes, worsening clearance of the aberrant protein aggregate (van der Kant et al., 2020). Normally, PS-1 binds to unglycosylated V_o_a1, priming its N-glycosylation by the oligosaccharyltransferase. N-glycosylation is required to efficiently deliver V_o_a1 from the ER to the lysosome, which acidifies and activates various enzymes important for autophagosomes and the clearance of protein aggregates (Lee et al., 2010). Thus, PS-1 is a common mechanism linking the dual pathogenic processes in AD; it increases Aβ formation and decreases Aβ aggregate clearance. It also highlights the importance of proper localization of V_o_a1 in the context of AD. Although the PS-1 variant is reported to be the most pathogenic variant in AD (Xiao et al., 2021), further studies are required to clarify the exact interaction between PS-1 and V_o_a1 of V-ATPase. One study reported impaired V_o_a1 N-glycosylation of PS-1 and lysosomal acidification in 5xFAD mice (Avrahami et al., 2013). However, another study found a contradictory result, with no change to V_o_a1 N-glycosylation in mouse embryonic fibroblast nor alteration in lysosomal and autophagic function (Zhang et al., 2012).

## 4. Diseases related to non-neural subunit a isoforms

### 4.1. Diseases related to *ATP6V0A2* mutations

Early mice studies reported a near-ubiquitous expression of subunit a2 mRNA (excluding skeletal muscle), with the strongest expression in the kidney and liver (Nishi and Forgac, 2000). In humans, a2 is encoded by *ATP6V0A2* on chromosome 12q.24.3, consisting of 21 exons (Morava et al., 2009). Mutations leading to the loss of *ATP6V0A2* gene function and impaired a2 production are responsible for autosomal recessive cutis laxa type II (ARCL II), which is a distinct type of the congenital disorders of glycosylation (CDG) (Kornak et al., 2008; Morava et al., 2009). ARCL is a connective tissue disorder divided into three types, with clinical features including loose and inelastic skin shared among all three. Specifically, the ARCL II phenotype includes developmental delays, skeletal abnormalities, a variable severity of cutis laxa (loose skin), and neurological abnormalities such as epilepsy, cognitive delay, and mental deterioration (Morava et al., 2009). The phenotypic spectrum of cutis laxa ranges from mild wrinkly skin syndrome (WSS) to the more severe Debré-type cutis laxa (Kornak et al., 2008). Individuals with Debré-type cutis laxa displayed more neurological disorders relative to WSS patients (Maldergem et al., 2023). Patients with ARCL II arising from *ATP6V0A2* also have defects in N- and O-glycosylation and sialylation, giving the disease its CDG classification (Kornak et al., 2008; Morava et al., 2008).

Glycosylation occurs at the endoplasmic reticulum and the Golgi to modify protein structure, function, and stability (Reily et al., 2019). This process is highly dependent on compartment-specific enzyme activity. The a2 subunit displays Golgi-localization in the cell type responsible for ARCL II manifestations: the human dermal fibroblast (Fischer et al., 2012). The pathophysiology of ARCL II arising from *ATP6V0A2* mutations comes from V-ATPase pumps’ failure to maintain proper pH in the Golgi for optimum glycosyltransferase activity, thus impairing glycosylation and organelle transport (Kornak et al., 2008; Morava et al., 2008; Rivinoja et al., 2009). This leads to abnormal elastin synthesis as tropoelastin accumulates inside the cell and prevents mature elastin transport to the extracellular space, creating the deleterious cutaneous phenotype observed in ARCL II (Hucthagowder et al., 2009; Shafagh Shishavan and Morovvati, 2022). The neurological abnormalities associated with ARCL II are caused by alterations to neurometabolic homeostasis initiated by aberrant glycosylation (Hucthagowder et al., 2009; Callewaert and Urban, 2022). As a result, the processing and secretion of many brain proteins is compromised.

There are various *ATP6V0A2*-associated mutations that manifest as ARCL II, including the homozygous R63X and Q765X nonsense mutations (Kornak et al., 2008). These mutations are unique as they have been found in several patients who differ geographically and genetically (Hucthagowder et al., 2009; Bahena-Bahena et al., 2014). The two mutations also have lower mRNA expression and a truncated protein product, compromising V-ATPase function and leading to glycosylation and neurological defects (Kornak et al., 2008). Moreover, the heterozygous L811P missense mutation is located on the last α-helix and changes the helix conformation, thereby disturbing proton translocation and subunit a2 interactions with other subunits (Fischer et al., 2012). However, the heterozygous L811P mutation alone likely does not cause ARCL II and forms a somewhat stable protein. Nevertheless, L811P exists as a compound heterozygous mutation along with the splicing mutation c.2055 + 2dupT, which caused the associated disease (Fischer et al., 2012). Lastly, the homozygous P405L missense mutation is located in the first α-helix and is a well-studied ARCL II-causing mutation (Hucthagowder et al., 2009; Esmail et al., 2018a). Interestingly, the P405L subunit a4 is N-glycosylated considering ARCL II is characterized by defective N-glycosylation (Esmail et al., 2018a). Despite this, the P405L variant experiences high degradation rates, so the authors proposed that P405 is required for complex assembly, stability, and Golgi trafficking (Esmail et al., 2018a).

**Figure 5:**
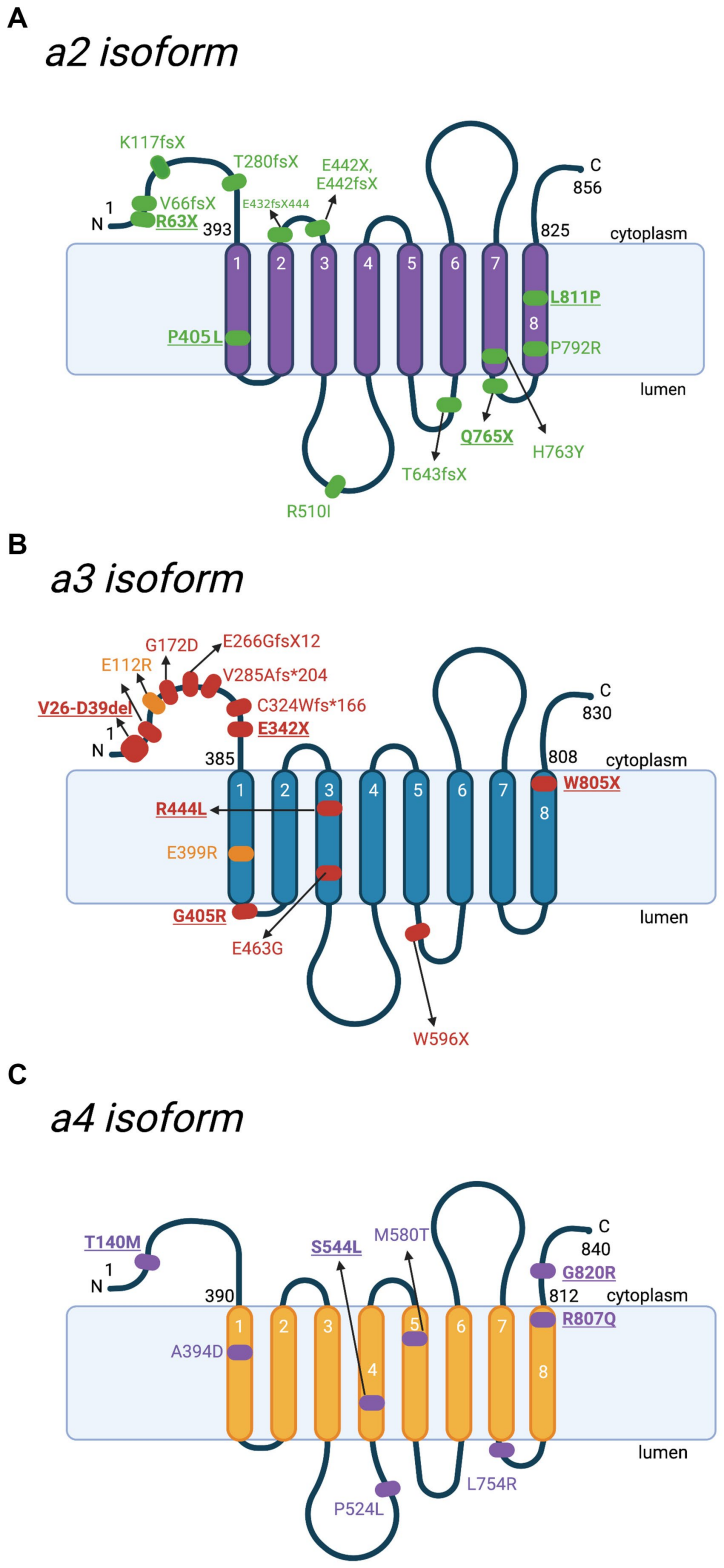
Schematic representation of a2-a4 subunits and location of mutations with the corresponding diseases. Besides a1, there are 3 other isoforms of V-ATPase subunit a. Various mutations have been reported in different diseases. Key mutations that are heavily discussed in the text are bolded and underlined. **(A)** Locations of various mutations to the a2 subunit, identified in cases of autosomal recessive cutis laxa type II (ARCL II). Amino acid sequences are based on Q9Y487 in UniProt. **(B)** Locations of identified mutations to the a3 subunit contributing to malignant infantile osteopetrosis (MIOP) (red) and its milder form (yellow). Amino acid sequences are based on Q13488 in UniProt. **(C)** Locations of mutations to the a4 subunit involved in distal renal tubular acidosis (dRTA). Amino acid sequences are based on Q9HBG4 in UniProt. For the purpose of annotation, transmembrane helices α_7_-α_8_ are not tilted. The figure was created with BioRender.com.

Some other early reported *ATP6V0A2-*causing ARCL II variants include the frameshift mutations K117fsX144 and E442fsX506, along with the nonsense mutation E442X (Figure 5A; Kornak et al., 2008). Kornak et al. (2008) also reported *ATP6V0A2* frameshift mutations present in individuals with WSS: V66fsX107 and T643fsX683. Furthermore, the missense mutations R510I and P792R, the heterozygous frameshift variant E432fsX444, and the compound heterozygous mutation: H763Y and c.1326 + 1G > A are *ATP6V0A2-*causing ARCL II variants (Hucthagowder et al., 2009; Fischer et al., 2012).

### 4.2. Diseases related to *ATP6V0A3* mutations

The *ATP6V0A3* gene on chromosome 11q13 encodes the human a3 subunit (gene is also known as *TCIRG1*, *Atp6i*, or *OC116*) (Heaney et al., 1998; Heinemann et al., 1999). The gene transcript encodes both OC116 in the osteoclast and T-cell Immune Response cDNA 7 (TIRC7) protein ubiquitously. In mice, the *ATP6V0A3* transcript is strongly expressed in the liver and heart (Nishi and Forgac, 2000). Additionally, osteoclast differentiation induces a3 expression on the plasma membrane (Toyomura et al., 2000). Osteoclasts are cells responsible for bone degradation through the acidification of a sealed extracellular interface, ultimately regulated by the V-ATPase (Kornak et al., 2000). V-ATPases are found in the ruffled membrane borders, pumping protons to maintain a low pH, which is crucial for dissolving inorganic bone material and promoting the activity of proteases that further degrades the bone matrix (Blair, 1998).

Mutations to the *ATP6V0A3* gene are the most common genetic cause of the rare autosomal recessive disease, malignant infantile osteopetrosis (MIOP), which manifests early in life and is fatal if left untreated (Kornak et al., 2000; Michigami et al., 2002). In this condition, osteoclasts fail to resorb bone and clinical symptoms include osteosclerosis, thrombocytopenia, anemia, hepatosplenomegaly, and in some cases, visual impairment (Kornak et al., 2000). Alteration to normal bone resorption and remodeling results in an abundance of osteoid, which reduces bone marrow space, a region where hematopoiesis occurs, leading to the gradual reduction of blood production (Vomero et al., 2019). In severe cases, patients show neurological complications, including cranial neuropathies, due to progressive compression of the cranial nerves, spinal cords, and blood vessels (Steward, 2003).

There are various mutations reported in MIOP patients, which lead to V-ATPase loss of function (Figure 5B; Table 1). The earliest study by Kornak et al. (2000) identified a biallelic splice site mutation of the *OC116* gene, V26-D39del, of the cytoplasmic V_o_ N-terminus. The N-terminus is crucial for forming the V_1_ complex and V-ATPase assembly *in-vivo*. Thus, mutations to this region often result in V-ATPase dysfunction (Liberman et al., 2013). Furthermore, protein truncation, due to premature stop codons at linker regions after the 3rd or 5th transmembrane domains (A480fsX or W596X), results in total loss of a3 function (Kornak et al., 2000). Two heterozygous stop mutations were also identified: E342 on the N-terminus and W804 on the C-terminus (W805 based on the updated accession number NM_006019.4), which is suspected to be in compound heterozygosity with other mutations yet to be discovered (Kornak et al., 2000). Substitution by an arginine in the G405R variant introduced a sterical hindrance for the 1^st^ vacuolar loop bending between the 1^st^ and 2^nd^ 𝛼-helices (Figure 5B; Table 1; Sobacchi et al., 2001; Susani et al., 2004). Substitution of the R444 residue in the 3^rd^ transmembrane 𝛼-helix with a hydrophobic leucine causes instability to the basic residue (Figure 5B; Table 1; Sobacchi et al., 2001).

Additionally, mutations on the splice donor site, such as a T-to-C transition on intron 19, a common region to *OC116* and *TIRC7*, potentially resulted in abnormal splicing of both transcripts (Michigami et al., 2002). Therefore, MIOP patients commonly presented immune anomalies and a high incidence of infections (Vomero et al., 2019). Some other reported variants in the *ATP6V0A3* are compound heterozygous mutations of E266GfsX12 with E463G (Vomero et al., 2019), E399RTer with E112R in a milder version of the disease (Luong et al., 2022), G172D, V285Afs*204, and c.C324Wfs*X166 (Figure 5B; Liu et al., 2021).

### 4.3. Disease related to *ATP6V0A4* mutations

The human a4 isoform is encoded by the *ATP6V0A4* gene located on chromosome 7q34 and is expressed in α-intercalated and β-intercalated cells of the kidneys, ocular ciliary bodies, pigmented epithelial cells of the retina, the human inner ear, and the epididymis (Oka et al., 2001a; Stover et al., 2002; Pietrement et al., 2006; Kawamura et al., 2010). *ATP6V0A4* was identified as an early causative gene of distal renal tubular acidosis (dRTA) along with the gene encoding subunit B1, *ATP6V1B1* (Karet et al., 1999; Smith et al., 2000; Stover et al., 2002). *ATP6V0A4* and *ATP6V1B1*-related dRTA account for ~50–60% of primary dRTA cases and display autosomal recessive inheritance (Wagner et al., 2023). Distal renal tubular acidosis is characterized by the inability to secrete protons in α-intercalated cells of the cortical and outer medullary collecting ducts (Wagner et al., 2023). This reduces urinary acidification and ammonium excretion and leads to acidosis. Clinically, dRTA patients develop a range of phenotypes arising directly from cellular defects caused by dRTA-related genes and the indirect effect of acidosis (Wagner et al., 2023). Some acidosis-related effects include rickets, hypokalaemia, nephrocalcinosis, and hypercalciuria (Caldas et al., 1992; Alexander et al., 2016; Vallés and Batlle, 2018). Hypokalaemia arises due to the altered balance between proton, potassium, and sodium transport (Sebastian et al., 1971; Batlle et al., 2006). Meanwhile, the increased acid in the blood causes bone to release calcium as a means of buffering. This, along with decreased calcium absorption due to acidosis, contributes to nephrocalcinosis and hypercalciuria (Alexander et al., 2016). Mutations in subunit a4 of the V-ATPase can lead to impaired V-ATPase trafficking, assembly, activity, or its interaction with other proteins, thereby affecting urine acidification and causing dRTA (Ochotny et al., 2006).

Inspired by two naturally occurring homozygous missense mutations in subunit a4, R807Q, and G820R, that cause dRTA, Su et al. (2008) recreated these mutations in yeast (Vph1p isoform) to explore them further (Figure 5C; Table 1). The two aforementioned residues lie within the phosphofructokinase-1-binding domain of a4, which is a key regulator of glycolysis (Su et al., 2008). The study revealed that the G820R mutant led to a complete loss of binding for phosphofructokinase-1, followed by the severe deterioration of proton translocation and the mild destruction of ATPase activity. The mild interruption of ATPase activity (36% loss) combined with the severe decline of proton translocation (78% loss) suggests that the a4-phosphofructokinase-1 interaction functionally couples the ATPase activity to proton transport (Su et al., 2008; Ghazi et al., 2020). Nevertheless, more recent 3D molecular modeling displayed that the G820R mutation forms a putative salt bridge with the negatively charged a4 Glu-729 residue, which is important in the proton translocation pathway (Toei et al., 2011; Esmail et al., 2018a). The formation of this salt bridge is believed to interfere with the proton channel structure and thus hinder proton translocation (Esmail et al., 2018a). The conformational change induced by the salt bridge may also contribute to the damaged a4-phosphofructokinase-1 interaction (Esmail et al., 2018a). Moreover, the R807Q mutation reduced subunit a4 production, leading to the severe loss of enzymatic function, but did not affect phosphofructokinase-1 binding (Su et al., 2008). R807 in a4 is a paralog to a1 R804, meaning it serves as a key residue to transport protons into the lumen (Su et al., 2008; Abbas et al., 2020). The significance of this conserved residue accounts for the severe R807Q mutation phenotype observed.

The first known heterozygous mutation in the a4 subunit (S554L), causing incomplete distal renal tubular acidosis, was identified in a 40-year-old man (Imai et al., 2016). Clinical features included urinary proton excretion dysfunction causing excess loss of potassium instead of proton, along with hypokalaemia and nephrocalcinosis. However, metabolic acidosis was absent. The same heterozygous missense mutation was later observed in four related patients who exhibited complete dRTA (Chen et al., 2020). Transfected HEK293T cells carrying the S544L mutation also exhibited hindered ATPase activity and the inability to bind subunit B1, which may affect V-ATPase assembly (Chen et al., 2020). This finding is interesting and warrants further investigation, as this is the only missense mutation from a pool of over 40 a4 mutations where dRTA is inherited in a dominant manner (Su et al., 2008; Pereira et al., 2015; Gómez et al., 2016; Imai et al., 2016; Chen et al., 2020). Another notable *ATP6V0A4*-related dRTA mutation includes a patient with a digenic inheritance of dRTA, being heterozygous for the *ATP6V1B1* (D146G) and *ATP6V0A4* (T140M) missense mutations (Nagara et al., 2018). This is the first observed digenic inheritance of dRTA and helps increase our understanding of disease inheritance and genetic diagnostics (Nagara et al., 2018). Some other *ATP6V0A4*-associated dRTA mutations include the homozygous missense variants A394D, P524L, M580T, and L754R (Figure 5C; Smith et al., 2000; Gao et al., 2014; Park et al., 2018).

Fortunately, dRTA is a treatable disease, with the main line of treatment consisting of alkali supplementation (Lopez-Garcia et al., 2019). The alkali agents reduce acidosis to maintain acid–base homeostasis, which leads to the reduction of various symptoms. However, sensorineural hearing impairments, which are commonly associated with *ATP6V0A4*-associated dRTA, cannot be treated by alkali supplementation (Wagner et al., 2023). *ATP6V0A4* is highly expressed in the marginal cells in the stria vascularis, which is needed for regulating endolymph pH in the cochlea of the inner ear (Stanković et al., 1997). *ATP6V0A4* mutations affecting V-ATPase function may alkalinize the cochlear endolymph, leading to sensorineural deafness (Stover et al., 2002; Lopez-Garcia et al., 2019). Therefore, treating *ATP6V0A4*-associated dRTA with alkali manages the symptoms of acidosis, not the genetic manifestation of hearing loss.

## 5. Conclusion

The V-ATPase is an ATP-dependent proton pump that holds a prominent housekeeping role in maintaining characteristic acidic pH for homeostatic purposes and, eventually, organism survival. It is present in both plasma and intracellular organelle membranes and is a multisubunit protein complex that operates by a rotary mechanism, coupling ATP hydrolysis at the cytosolic domain (V_1_) with proton translocation at the membrane-embedded domain (V_o_). Recently, the structures of human and other mammalian V-ATPases have been uncovered via cryo-electron microscopy (Abbas et al., 2020; Wang L. et al., 2020; Wang R. et al., 2020). Elucidating these structures is imperative to understand the functional and structural consequences of various V-ATPase subunit mutations for disease pathophysiology and treatment. Moreover, as a multisubunit structure, the V-ATPase exhibits cell-specific subunit isoforms regulating its localization and specialized function. Aberrant mutations to any subunits have been associated with various human diseases. Mutations to subunit a1 are involved in neurodevelopmental disorders, namely PME and DEE. Mutations to isoform a2 result in glycosylation impairments, leading to the connective tissue disorder ARCL II and a3 variants are the leading cause of MIOP. Finally, isoform a4 mutations often cause dRTA and sensorineural hearing loss.

So far, studies have evaluated the effect of V_o_a1 mutations on lysosomal acidification and autophagic function in neurodevelopment (Aoto et al., 2021; Bott et al., 2021). However, their animal models did not display epileptic events, which are the common symptoms of DEE and PME. Conversely, a previous study reported that a conditional knockout of V_o_a1 in the mouse hippocampus resulted in hyperexcitability of the CA3 network, which manifested as nonconvulsive electrographic seizures events in the electroencephalogram (Ma et al., 2019). Thus, further investigations looking specifically at how such mutations resulted in a seizure phenotype would help understand the impact of V_o_a1 mutations on neuronal electrical firing diseases, propelling the advancement in treatment options, including precision therapy. It is also possible that the resulting symptoms in DEE and PME come from a complex interaction of multiple mutated genes. Consequently, it is imperative to inspect the interactions of multiple genes in DEE and PME pathophysiology for potential polygenic inheritance mode. Examining relations between epilepsy and developmental impairment in *ATP6V0A1* variants is also instrumental in the treatment plan, whether seizure management would help dampen neurodevelopmental impairment or if the two are separated.

Overall, recent findings on *ATP6V0A1* mutations in neurodevelopmental disorders emphasize the paramount role of V-ATPases in the development and maintenance of proper lysosomal and autophagosomal activity. In light of these growing discoveries, further structural and functional investigation of human subunit a1 mutations and other isoforms will be beneficial to help elucidate their pathophysiology for disease treatment.

## Author contributions

SS conceived and designed the manuscript. KI and PA drafted and wrote the manuscript. All authors contributed to the article and approved the submitted version.

## Funding

This work was supported by the Natural Sciences and Engineering Research Council of Canada (RGPIN 2020 07139) and the Canadian Institute of Health Research (CIHR PJT 165917).

## Conflict of interest

The authors declare that the research was conducted in the absence of any commercial or financial relationships that could be construed as a potential conflict of interest.

## Publisher’s note

All claims expressed in this article are solely those of the authors and do not necessarily represent those of their affiliated organizations, or those of the publisher, the editors and the reviewers. Any product that may be evaluated in this article, or claim that may be made by its manufacturer, is not guaranteed or endorsed by the publisher.
